# Job Crafting Through the Lens of Paradoxical Leadership: The Role of Positive Psychological Capital and Promotive Voice

**DOI:** 10.3390/bs16060844

**Published:** 2026-05-25

**Authors:** Yueying Wang, Jiaming Hu, MyeongCheol Choi, Hann Earl Kim

**Affiliations:** 1 School of Business, Xijing University, Xi’an 710123, China; 202350022@gachon.ac.kr; 2 Department of Business Management, Gachon University, Seongnam 13120, Republic of Korea; hk3624@gachon.ac.kr

**Keywords:** paradoxical leadership, positive psychological capital, promotive voice, job crafting

## Abstract

This study examines the relationships among paradoxical leadership, positive psychological capital, promotive voice, and job crafting. This study proposes and tests a mediation framework in which positive psychological capital and promotive voice link paradoxical leadership to job crafting. Higher levels of positive psychological capital, comprising hope, efficacy, resilience, and optimism, in turn stimulate promotive voice, which encourages employees to proactively reshape their jobs. Paradoxical leadership and job crafting are conceptually connected through their shared emphasis on navigating complexity, flexibility, and proactive adaptation in contemporary organizations. Empirical evidence indicates that paradoxical leadership is positively associated with positive psychological capital and job crafting, and that both positive psychological capital and promotive voice independently mediate the relationship between paradoxical leadership and job crafting. These findings reveal that paradoxical leadership is linked to job crafting not only by strengthening employees’ internal psychological resources but also by encouraging proactive voice behavior. Therefore, investigating the relationship between paradoxical leadership and job crafting not only advances leadership, but also offers actionable insights for organizations seeking to enhance flexibility, innovation, and sustainable performance through leadership practices that empower employees to actively craft their jobs.

## 1. Introduction

Contemporary organizations increasingly operate in environments characterized by complexity, uncertainty, and competing demands. In such contexts, employees are expected not only to fulfill formal job requirements but also to proactively adapt to changing organizational expectations and work conditions (Li et al., 2025; Xia et al., 2026). As organizations strive to remain agile and competitive, employees are increasingly encouraged to modify and redefine aspects of their work roles in ways that align with both organizational goals and personal development needs (Tims & Bakker, 2010; Bakker, 2017). This proactive and self-initiated behavior, commonly referred to as job crafting, has attracted growing attention in organizational behavior research because it enables employees to better cope with changing work demands while enhancing engagement, adaptability, and effectiveness.

Given the growing importance of employee adaptability, researchers have increasingly emphasized the role of leadership in shaping proactive work behaviors. Among emerging leadership approaches, paradoxical leadership has gained considerable attention due to its emphasis on integrating seemingly contradictory yet interdependent leadership behaviors (Smith & Lewis, 2011; Y. Zhang et al., 2015; K. Zhang et al., 2025). Rather than treating organizational tensions as mutually exclusive, paradoxical leaders attempt to simultaneously balance competing demands, such as maintaining control while granting autonomy, ensuring consistency while allowing flexibility, and preserving both closeness and professional distance (Lewis et al., 2014). Through this balanced approach, paradoxical leadership may help employees interpret contradictory organizational expectations, respond more effectively to uncertainty, and adapt more proactively to evolving work environments.

Recent studies suggest that paradoxical leadership may play an important role in encouraging employee adaptability and proactive behaviors (Khan et al., 2025; Devi, 2024). By simultaneously providing structure and flexibility, paradoxical leaders may create work environments that encourage employees to actively reshape and optimize their work roles. In this regard, paradoxical leadership may be positively associated with job crafting because employees are better able to reconcile competing demands, identify opportunities for improvement, and engage in self-initiated work adjustments (Bindl et al., 2019; Karimi et al., 2023). However, despite increasing scholarly interest in paradoxical leadership, the underlying mechanisms through which paradoxical leadership influences employees’ job crafting behaviors remain insufficiently understood.

To address this gap, the present study examines two important explanatory mechanisms linking paradoxical leadership and job crafting. First, positive psychological capital, which reflects employees’ positive psychological resources such as self-efficacy, optimism, hope, and resilience (Toor & Ofori, 2010), may function as a critical psychological pathway through which paradoxical leadership enhances employee adaptability (Ahmed & Khan, 2024; Tan et al., 2024). Employees with stronger psychological resources are generally more likely to proactively modify and improve their work roles in response to changing work demands (Kamil et al., 2024). Second, promotive voice, defined as employees’ constructive expression of improvement-oriented suggestions (Kataria & Varma, 2025), may serve as an important behavioral mechanism connecting leadership and job crafting. Employees with higher levels of psychological capital are more likely to express promotive voice and actively participate in improving work processes (Rego et al., 2012; Agarwal & Farndale, 2017), which may further encourage them to engage in job crafting behaviors.

Based on these arguments, this study proposes that positive psychological capital and promotive voice function as important mechanisms linking paradoxical leadership and job crafting. Specifically, paradoxical leadership may strengthen employees’ psychological resources and encourage constructive voice behaviors, which subsequently facilitate job crafting. By clarifying these psychological and behavioral pathways, this study contributes to the paradoxical leadership and job crafting literature and provides a more integrated understanding of how leadership influences employee adaptation in complex organizational environments. Furthermore, the findings offer practical implications for organizations seeking to cultivate a more adaptive, resilient, and engaged workforce.

## 2. Theory and Hypotheses

### 2.1. Paradoxical Leadership and Positive Psychological Capital

Paradoxical leadership, which involves integrating and managing competing demands, has attracted increasing attention because of its potential to foster positive employee outcomes in complex organizational environments (Khan et al., 2025; Liu et al., 2020). Positive psychological capital refers to employees’ positive psychological resources, including self-efficacy, optimism, hope, and resilience, which collectively reflect an individual’ s capacity to adapt and function effectively under challenging conditions (Rabenu, 2017). In organizational contexts characterized by uncertainty and conflicting expectations, paradoxical leaders may help employees interpret tensions more constructively by simultaneously providing flexibility, support, guidance, and performance expectations (Smith & Lewis, 2012; Stynen & Semeijn, 2023).

By balancing seemingly contradictory leadership behaviors, paradoxical leaders may strengthen employees’ confidence in managing complex work demands and coping with uncertainty (Jung et al., 2022). Leaders who effectively integrate stability and flexibility may encourage employees to maintain positive expectations toward their work and remain psychologically resilient when facing setbacks or organizational change (Huang et al., 2022; Ahmed & Khan, 2024). In addition, the combination of support and challenge embedded in paradoxical leadership may help employees develop stronger psychological resources and adaptive capacities (Franken et al., 2020; Yin, 2022). As employees observe leaders managing tensions in a constructive manner, they may become more psychologically prepared to respond positively to workplace challenges (Akhtar & Riaz, 2024).

Taken together, paradoxical leadership may contribute to the development of positive psychological capital by fostering employees’ confidence, optimism, resilience, and adaptive capabilities (Devi, 2024). Employees with stronger psychological capital are more likely to maintain positive attitudes, cope effectively with difficulties, and engage actively in constructive work behaviors. Therefore, the following hypothesis is proposed:

**Hypothesis** **1.** *Paradoxical leadership has a positive effect on positive psychological capital*.

### 2.2. Paradoxical Leadership and Job Crafting

Paradoxical leadership is associated with job crafting because it creates a work environment that encourages employees to take initiative and adapt their work roles in response to changing demands (Akhtar & Riaz, 2024). Job crafting refers to employees’ proactive behavioral modifications of their work tasks and relational boundaries. In this study, we focus on behavioral job crafting, emphasizing employees’ actual changes to work activities rather than purely cognitive reframing or avoidance-oriented crafting (Bakker et al., 2012). By balancing autonomy and structure, paradoxical leaders may provide employees with both flexibility and guidance, enabling them to reshape their work roles while remaining aligned with organizational goals (Tan et al., 2024).

Paradoxical leaders may encourage job crafting by fostering a work climate characterized by adaptability, support, and reduced uncertainty (Jerab & Mabrouk, 2023). Leaders who simultaneously emphasize stability and flexibility may help employees feel more confident in experimenting with alternative approaches to their work and adjusting their tasks to better fit evolving job demands (Fürstenberg et al., 2021; Park et al., 2022). In addition, paradoxical leadership may strengthen employees’ sense of psychological safety by helping them interpret competing expectations more constructively and reducing concerns about interpersonal risk (Kim, 2021). As employees feel supported in navigating complex work environments, they may become more willing to proactively modify and redefine aspects of their work roles (Pradies et al., 2021).

Taken together, paradoxical leadership may facilitate job crafting by encouraging employees to respond more actively and adaptively to workplace challenges. By providing both support and flexibility, paradoxical leaders create conditions that enable employees to reshape their work experiences in ways that benefit both individual development and organizational effectiveness. Accordingly, the following hypothesis is proposed:

**Hypothesis** **2.** *Paradoxical leadership has a positive effect on job crafting*.

### 2.3. Positive Psychological Capital and Promotive Voice

Positive psychological capital has been recognized as an important psychological resource that encourages employees to engage in constructive and proactive workplace behaviors (De Waal & Pienaar, 2013). Promotive voice refers to employees’ expression of improvement-oriented suggestions intended to enhance organizational functioning. Employees with higher levels of positive psychological capital are generally more confident, optimistic, and resilient when responding to workplace challenges, which may increase their willingness to express constructive ideas and participate actively in organizational improvement (Luc, 2023).

From the perspective of conservation of resources theory, employees with stronger psychological resources are more capable of mobilizing and maintaining personal resources when facing uncertainty or interpersonal risk (Chen & Li, 2026; Li & Song, 2024; Sweetman & Luthans, 2010). As a result, they may feel more comfortable expressing suggestions and proposing changes aimed at improving work processes and organizational effectiveness. In particular, employees with higher psychological capital are more likely to believe that their input is meaningful and capable of generating positive outcomes, thereby increasing their willingness to engage in promotive voice behaviors (Farndale et al., 2011). In addition, optimistic and resilient employees may be more likely to persist in expressing constructive opinions even when encountering resistance or setbacks (Avey et al., 2008).

Taken together, positive psychological capital may function as an important antecedent of promotive voice by strengthening employees’ confidence, initiative, and willingness to contribute to organizational improvement. Employees with stronger psychological resources are more likely to communicate constructive suggestions and engage actively in improvement-oriented behaviors. Accordingly, the following hypothesis is proposed:

**Hypothesis** **3.** *Positive psychological capital has a positive effect on promotive voice*.

### 2.4. Promotive Voice and Job Crafting

Promotive voice refers to employees’ constructive expression of suggestions aimed at improving organizational functioning (Kataria & Varma, 2025). Job crafting refers to employees’ proactive behavioral modifications of their work tasks and work boundaries to better align with their interests, abilities, and work demands (F. Zhang & Parker, 2019). Employees who actively express improvement-oriented ideas may become more aware of opportunities to adjust and improve their own work roles, thereby increasing their likelihood of engaging in job crafting behaviors (Wang, 2021).

When employees communicate constructive suggestions and participate actively in organizational improvement, they may develop a stronger sense of ownership and involvement in their work processes (Bakker, 2010; Adelman, 2012). This increased involvement may encourage employees to proactively modify aspects of their work in ways that improve job fit, satisfaction, and effectiveness (Li & Jo, 2025). In addition, promotive voice may foster a supportive and participative work environment in which employees feel more comfortable experimenting with new approaches to their tasks and responsibilities (Curcuruto & Griffin, 2023). As employees become more engaged in improving work processes, they may also become more willing to reshape their own work activities and role boundaries (Judge et al., 2023).

Taken together, promotive voice may function as an important antecedent of job crafting by encouraging employees to participate actively in organizational improvement and proactively adjust their work roles. Employees who express constructive suggestions are more likely to engage in behavioral changes that improve the alignment between their work and personal or organizational goals. Accordingly, the following hypothesis is proposed:

**Hypothesis** **4.** *Promotive voice has a positive effect on job crafting*.

### 2.5. Mediating Effect of Positive Psychological Capital

Positive psychological capital may explain how paradoxical leadership influences employees’ job crafting behaviors. Paradoxical leadership creates a work environment characterized by flexibility, support, and the effective management of competing demands, which may strengthen employees’ psychological resources and adaptive capacities (Tan et al., 2024). As employees become more psychologically confident and resilient in responding to complex work situations, they may become more willing to proactively modify and reshape their work roles (Hussein & Amiruddin, 2020).

Paradoxical leadership may foster employees’ self-efficacy, optimism, hope, and resilience by helping them interpret workplace challenges more constructively and adapt effectively to changing organizational conditions (Yin, 2022). Employees with stronger positive psychological capital are more likely to believe that they can successfully influence their work environment and cope with evolving job demands (Iqbal et al., 2024; Shah et al., 2019). These psychological resources may subsequently encourage employees to engage more actively in job crafting behaviors by increasing their confidence and motivation to initiate changes in their work activities (Franken et al., 2020).

Taken together, positive psychological capital may function as an important psychological mechanism through which paradoxical leadership promotes job crafting. By strengthening employees’ psychological resources and adaptive capacities, paradoxical leadership may indirectly encourage proactive changes in employees’ work roles. Accordingly, the following hypothesis is proposed:

**Hypothesis** **5.** *Positive psychological capital mediates the relationship between paradoxical leadership and job crafting*.

### 2.6. Mediating Effect of Promotive Voice

Promotive voice may function as an important behavioral mechanism linking paradoxical leadership and job crafting. Paradoxical leadership encourages employees to navigate competing demands and adapt to complex organizational environments, which may increase employees’ willingness to express constructive suggestions aimed at improving work processes and organizational functioning (Lavine, 2014). Through promotive voice, employees may become more actively involved in organizational improvement and more aware of opportunities to reshape their own work roles (Ajmal et al., 2025).

When leaders balance flexibility and structure while encouraging open communication, employees may feel more comfortable expressing ideas and participating in constructive change-oriented behaviors (Khan & Ullah, 2025; Mansaray, 2019; Qiu et al., 2025). Employees who engage in promotive voice are more likely to develop a stronger sense of involvement and ownership toward their work, which may subsequently encourage them to proactively adjust their work activities and role boundaries (Akhtar & Riaz, 2024). As employees become more engaged in proposing improvements and participating in workplace change, they may also become more willing to implement proactive modifications to their own work roles (Schaufeli, 2021).

Taken together, promotive voice may serve as a behavioral pathway through which paradoxical leadership influences job crafting. By encouraging constructive voice behaviors and active participation in organizational improvement, paradoxical leadership may indirectly promote employees’ proactive role modification and work adaptation. Accordingly, the following hypothesis is proposed:

**Hypothesis** **6.** *Promotive voice mediates the relationship between paradoxical leadership and job crafting*.

**Figure 1:**
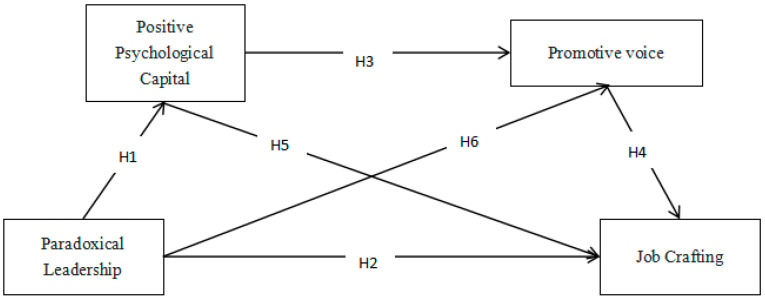
Research model. Note. H5 and H6 denote hypothesized mediation paths that form part of the indirect effects rather than standalone direct relationships.

These hypotheses form the basis of our conceptual model (Figure 1).

## 3. Methods

### 3.1. Sample and Data Collection

To explore the impact of paradoxical leadership on positive psychological capital and employee voice capabilities, this study collected data through questionnaires distributed via a widely used academic survey platform. Participation was entirely voluntary and anonymous. The survey was conducted from 15 July 2025 to 15 September 2025. A total of 400 online questionnaires were distributed, of which 344 valid responses were obtained, resulting in an effective response rate of 86%. The sample consisted of 54.7% male and 45.3% female respondents, indicating a relatively balanced gender distribution. Most participants were between 26 and 30 years old (28.2%) and 31–35 years old (35.2%), representing a critical stage of career development. Regarding education level, the sample primarily consisted of individuals with an undergraduate degree (55.2%), followed by those holding a master’s degree (16.6%), an associate degree (17.2%), a doctoral degree (4.1%), and a high school diploma or below (7.0%). In this study, an associate degree refers to a junior college or post-secondary vocational qualification below the bachelor’s level, whereas an undergraduate degree refers to a bachelor’s degree awarded by a university. With respect to work experience, most respondents reported 1–5 years (31.7%) or 6–10 years (43.0%) of tenure, indicating relatively rich professional experience. In terms of job position, the sample included ordinary employees (32.3%), first-line managers (31.1%), and middle-level managers (29.9%). Company size was also relatively evenly distributed: firms with fewer than 50 employees accounted for 9.0%, those with 51–100 employees for 25.9%, 101–200 employees for 23.5%, 201–500 employees for 24.1%, and more than 500 employees for 17.4%.

### 3.2. Measures

In this study, the initial scales were developed in English and then translated into Chinese using the back-translation method recommended by Brislin (1980). All constructs were measured using 5-point Likert scales ranging from 1 (strongly disagree) to 5 (strongly agree). For all measures, higher scores indicate higher levels of the corresponding constructs. Where necessary, items were reverse-coded prior to analysis.

#### 3.2.1. Paradoxical Leadership

Paradoxical Leadership (PL). This study uses a 14-item scale adapted from the original 22-item measure developed by Y. Zhang et al. (2015). The selected items were chosen to represent the five core dimensions of paradoxical leadership originally proposed by Y. Zhang et al. (2015). The response scale ranges from 5 (strongly disagree) to 1 (strongly agree). Example items are “The leader is able to meet the diverse needs of different teams within the organization simultaneously,” “The leader can find a balance amid contradictions,” and “The leader can maintain control while granting employees freedom.”

#### 3.2.2. Positive Psychological Capital

Positive Psychological Capital (PPC). This study uses an 8-item scale developed by Luthans et al. (2007). Example items are “I am confident in helping to solve problems at work,” “I am confident representing my work area in meetings with management,” and “I can think of many ways to achieve my current work goals.”

#### 3.2.3. Promotive Voice

Promotive Voice (PV). This study uses an 8-item scale developed by Liang et al. (2012). Example items are “I develop and make suggestions concerning issues that affect the work group,” “I express opinions about work-related issues even if they differ from others,” and “I actively encourage others to participate in improving the work environment.”

#### 3.2.4. Job Crafting

Job Crafting (JC). This study uses a 7-item scale developed by Tims et al. (2012). Example items are “I adjust the boundaries of my job to better fit my interests,” “I increase interactions with colleagues to enhance social relationships at work,” and “I change the way I complete my tasks to make them more efficient.”

## 4. Results

### 4.1. Common Method Bias

Considering that the scales used in this study are all self-report measures, Harman’s single-factor test was conducted to assess the potential presence of common method bias. The results showed that, in the full sample, the first factor explained 22.31% of the total variance. Similarly, this proportion was 15.52%, 27.23%, 14.41%, and 19.68% for the groups of paradoxical leadership, positive psychological capital, job crafting, and promotive voice, respectively. As all these values are well below the commonly accepted threshold of 50%, the results suggest that common method bias is unlikely to pose a serious concern in the present study.

### 4.2. Measurement Reliability and Validity Assessment

Table 1 presents the results of the confirmatory factor analysis (CFA) conducted using AMOS 26.0 on a sample of 340 respondents. The standardized factor loadings for all observed variables ranged from 0.668 to 0.820, exceeding the commonly accepted threshold of 0.60, thus indicating adequate indicator reliability. Construct reliability and convergent validity were assessed via Composite Reliability (CR) and Average Variance Extracted (AVE), respectively. The AVE values ranged between 0.521 and 0.626, surpassing the recommended cutoff of 0.50, which confirms sufficient convergent validity. Correspondingly, the CR values ranged from 0.735 to 0.883, exceeding the 0.700 benchmark, demonstrating satisfactory internal consistency of the latent constructs. In addition, Cronbach’s alpha coefficients were calculated for all constructs. The results indicated high internal consistency, with alpha values of 0.938 for paradoxical leadership, 0.899 for positive psychological capital, 0.847 for job crafting, and 0.931 for promotive voice. Regarding model fit, multiple indices indicated an excellent fit between the measurement model and the observed data. The chi-square to degrees of freedom ratio (χ^2^/df) was 1.131, below the acceptable threshold of 3.0. Incremental fit indices including CFI (0.990), TLI (0.989), IFI (0.990), and NFI (0.918) all surpassed the recommended value of 0.90. The Goodness of Fit Index (GFI) was 0.908, and the Root Mean Square Residual (RMR) was 0.066. The RMSEA value was 0.020, indicating a close fit to the data (values less than 0.05 are considered excellent). As shown in Table 1, the hypothesized measurement model demonstrates satisfactory and superior fit indices, indicating that it provides the best representation of the data among plausible model specifications. Taken together, these results provide strong empirical support for the reliability and validity of the measurement model, confirming its suitability for subsequent structural analyses.

To further examine the discriminant validity of the study constructs, a series of competing measurement models were tested and compared using confirmatory factor analysis. As shown in Table 2, the hypothesized four-factor model (paradoxical leadership, positive psychological capital, job crafting, and promotive voice) demonstrated the best overall model fit (χ^2^ = 301.126, df = 264, χ^2^/df = 1.131, NFI = 0.918, IFI = 0.990, TLI = 0.989, RMSEA = 0.020).

In comparison, alternative models showed progressively poorer fit indices. The three-factor model combining job crafting and promotive voice exhibited reduced fit (χ^2^/df = 1.354, RMSEA = 0.032), while the two-factor model further integrating positive psychological capital showed a noticeable deterioration in model fit (χ^2^/df = 2.056, RMSEA = 0.056). The one-factor model, in which all items loaded onto a single latent construct, demonstrated the poorest fit (χ^2^/df = 3.517, RMSEA = 0.086).

Chi-square difference tests indicated that the hypothesized four-factor model significantly improved model fit compared with the three-factor, two-factor, and one-factor models (all Δχ^2^ significant at *p* < 0.001). These results provide strong evidence for the discriminant validity of the four constructs and support the adequacy of the proposed measurement model.

### 4.3. Hypothesis Testing

Table 3 presents the descriptive statistics and Pearson correlation coefficients among the key study variables: paradoxical leadership (PL), positive psychological capital (PPC), job crafting (JC), and promotive voice (PV). The sample size for this analysis was 340. The mean scores for paradoxical leadership, positive psychological capital, job crafting, and promotive voice were 3.12 (SD = 1.054), 3.215 (SD = 1.083), 3.83 (SD = 1.115), and 3.636 (SD = 1.162), respectively. The standard deviations indicate moderate variability across the variables. Correlation analysis revealed significant positive relationships among all variables at the *p* < 0.01 level. Specifically, paradoxical leadership was positively correlated with positive psychological capital (r = 0.586, *p* < 0.01), job crafting (r = 0.655, *p* < 0.01), and promotive voice (r = 0.646, *p* < 0.01). Positive psychological capital also showed strong positive correlations with job crafting (r = 0.653, *p* < 0.01) and promotive voice (r = 0.665, *p* < 0.01). Furthermore, job crafting and promotive voice were significantly positively correlated (r = 0.747, *p* < 0.01). These findings suggest robust interrelations among the constructs, supporting the theoretical framework of the study and providing preliminary evidence for the hypothesized associations.

Table 4 summarizes the structural equation modeling (SEM) results for hypotheses 1 to 4, tested with a sample of 340 participants. All hypothesized paths were statistically significant at the *p* < 0.001 level. Specifically, paradoxical leadership (PL) had a positive and significant effect on positive psychological capital (PPC) (β = 0.665, SE = 0.069, CR = 9.632, *p* < 0.001) and job crafting (JC) (β = 0.266, SE = 0.062, CR = 4.329, *p* < 0.001). Positive psychological capital significantly influenced promotive voice (PV) (β = 0.824, SE = 0.079, CR = 10.469, *p* < 0.001), and promotive voice also positively affected job crafting (β = 0.787, SE = 0.081, CR = 9.754, *p* < 0.001). The overall model demonstrated a good fit with the data, as indicated by multiple fit indices: χ^2^/df = 1.225, *p* < 0.001, CFI = 0.982, TLI = 0.981, IFI = 0.982, RFI = 0.904, NFI = 0.910, and RMSEA = 0.026. These results provide empirical support for the proposed direct relationships among the latent constructs within the theoretical framework.

Table 5 reports the regression results related to the proposed mediation framework, whereas Table 6 presents the bootstrap mediation analyses used to test the indirect effects. Table 5 summarizes four structural models examining the relationships among paradoxical leadership (PL), positive psychological capital (PPC), promotive voice (PV), and job crafting (JC), while controlling for demographic variables including gender, age, and position.

In Model 1, paradoxical leadership (PL) significantly predicts positive psychological capital (β = 0.690, *p* < 0.001), controlling for gender (β = 0.299), age (β = 0.045), and position (β = 0.214, *p* < 0.05). Model 2 indicates a significant direct effect of paradoxical leadership on promotive voice (β = 0.801, *p* < 0.001), with control variables showing smaller but positive effects. Model 3 and Model 4 examine the influence of paradoxical leadership, positive psychological capital, and promotive voice on job crafting (JC). Paradoxical leadership maintains a significant positive effect on job crafting (Model 3: β = 0.341, *p* < 0.001; Model 4: β = 0.780, *p* < 0.001). Positive psychological capital (β = 0.319, *p* < 0.001) and promotive voice (β = 0.530, *p* < 0.001) also show significant positive effects on job crafting in Models 3 and 4, respectively.

**Figure 2:**
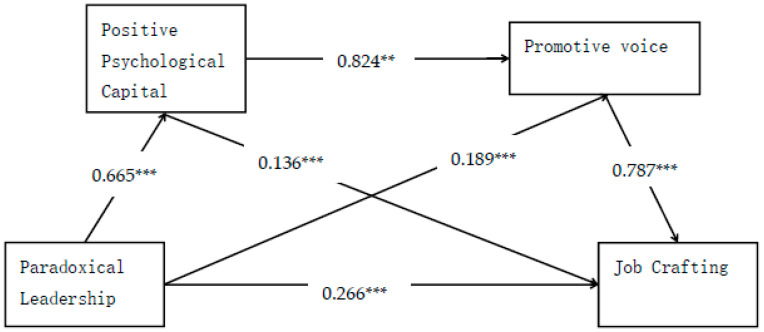
Path coefficient of variables. Note: ** *p* < 0.01, *** *p* < 0.001.

The R^2^ values indicate increasing explanatory power across the models: 0.357 and 0.425 for Models 1 and 2, respectively, and higher values of 0.636 and 0.431 for Models 3 and 4. The ΔR^2^ in Model 4 suggests a substantial increase in explained variance when both mediators are included. All F-values are significant at *p* < 0.001, indicating overall model significance. In addition, the bootstrap mediation results presented in Table 6 indicate that paradoxical leadership also exerts significant indirect effects on job crafting through positive psychological capital and promotive voice, suggesting that the overall relationship between paradoxical leadership and job crafting includes both direct and indirect pathways. These findings provide support for the mediating roles of positive psychological capital and promotive voice in the relationship between paradoxical leadership and job crafting (Figure 2).

Table 6 presents the results of the bootstrap mediation analysis. The total effect of PL on JC was 0.712 (95% CI [0.612, 0.808]), indicating that paradoxical leadership exerted a substantial overall influence on employees’ job crafting behaviors. In addition, the total indirect effect of paradoxical leadership on job crafting through the mediators was 0.446, with a 95% bootstrap confidence interval ranging from 0.368 to 0.530, indicating a significant overall indirect effect. These findings suggest that positive psychological capital and promotive voice play important explanatory roles in the relationship between paradoxical leadership and job crafting.

Regarding the specific indirect pathways, the indirect effect through positive psychological capital (PL → PPC → JC) was significant (effect = 0.136, 95% CI [0.076, 0.200]), accounting for 30.5% of the total indirect effect. The indirect effect through promotive voice (PL → PV → JC) was also significant (effect = 0.189, 95% CI [0.132, 0.258]), accounting for 42.4% of the total indirect effect. Furthermore, the serial indirect pathway through positive psychological capital and promotive voice (PL → PPC → PV → JC) was significant (effect = 0.121, 95% CI [0.086, 0.161]), accounting for 27.1% of the total indirect effect.

In addition to the indirect effects, paradoxical leadership retained a significant direct effect on job crafting after controlling for the mediating variables. Overall, these findings indicate that paradoxical leadership promotes employees’ job crafting both directly and indirectly by strengthening employees’ psychological resources and encouraging promotive voice behaviors, which subsequently facilitate proactive changes in employees’ work roles.

## 5. Discussion

The findings of this study offer important insights into how paradoxical leadership shapes employees’ job crafting. The results confirm that paradoxical leadership is positively associated with job crafting, indicating that when leaders effectively embrace and integrate competing expectations, employees are more likely to proactively redefine their work roles. This form of leadership fosters a dynamic and empowering environment that supports individuals in taking ownership of their tasks, relationships, and perceptions of work, encouraging a sense of responsibility and adaptability. By legitimizing diverse approaches to work, paradoxical leadership promotes a culture where innovation, personal initiative, and alignment between individual values and work activities are more likely to emerge.

The study also shows that positive psychological capital plays a significant mediating role in the relationship between paradoxical leadership and job crafting. Employees who perceive their leaders as capable of handling competing priorities in a constructive and consistent manner tend to develop stronger psychological resources, including hope, optimism, resilience, and self-efficacy. These psychological resources, as components of positive psychological capital, serve as internal motivational forces that inspire employees to approach their work with confidence and purpose. As individuals feel more capable of overcoming obstacles and pursuing meaningful goals, they become more inclined to reshape their job roles in ways that reflect both individual aspirations and organizational objectives (Bandura, 2023). The presence of these positive psychological states makes job crafting a more accessible and appealing strategy for employees seeking fulfillment and growth within their roles (Bakker, 2010). Furthermore, promotive voice emerges as another essential factor that bridges the link between leadership and job crafting. Employees who possess higher levels of psychological capital are more likely to express constructive suggestions aimed at improving work processes, team effectiveness, and overall organizational functioning (Newman et al., 2014). This tendency to speak up reflects a proactive orientation and a belief in one’s ability to contribute positively to the work environment. Through promotive voice, employees signal their engagement and commitment, which often translates into tangible efforts to reshape their work (Mowbray et al., 2024). As they articulate their insights and propose changes, they simultaneously engage in job crafting, seeking to enhance the meaning and effectiveness of their work through self-initiated adjustments.

Overall, the findings reveal a comprehensive process by which paradoxical leadership exerts influence on job crafting. Given the cross-sectional design of this study, the findings should be interpreted as evidence of associations among variables rather than definitive causal relationships Through the development of positive psychological states and the encouragement of open and constructive communication, this leadership style is associated with the presence of a psychologically supportive climate. These results contribute to a deeper understanding of how leadership approaches that value complexity and balance can promote not only organizational adaptability but also employee well-being and development. The study underscores the importance of viewing leadership as a catalyst for individual transformation and suggests that leaders who embrace paradoxes can effectively mobilize psychological and behavioral resources that support long-term organizational success.

## 6. Conclusions

### 6.1. Theoretical Implications

This research contributes to a deeper understanding of how leadership styles influence the motivation and performance of employees in contemporary organizational settings. Specifically, by identifying paradoxical leadership as a critical driver of both positive psychological capital and job crafting, this framework provides a nuanced contribution to the leadership literature. It moves beyond traditional models that often emphasize linear or one-dimensional leadership effects and instead emphasizes the dynamic and integrative nature of paradoxical leadership, which simultaneously promotes control and autonomy, uniformity and individualization. This approach sheds light on the capacity of such leadership to stimulate both the internal psychological growth of employees and their proactive engagement in reshaping their work roles. From a theoretical perspective, the model underscores the complex interplay between leader behaviors and employee development, positioning paradoxical leadership as a vital antecedent to positive psychological states that enable adaptive work behavior.

Moreover, the research incorporates positive psychological capital as an internal resource that links leadership with promotive voice and job crafting. This highlights the central role of psychological resources in the translation of leadership influence into proactive work behaviors. Rather than treating psychological capital as a static trait, the research conceptualizes it as a malleable outcome of leadership practices, thereby broadening the theoretical understanding of how employee strengths such as positive psychological capital can be cultivated through specific leader behaviors. The research also introduces promotive voice as a key behavioral mechanism through which internal psychological states are externalized in the workplace. This inclusion deepens the theoretical insight into how employee expression and suggestion-making behavior serve as essential intermediaries that connect internal motivation with changes in work roles. Together, the integration of positive psychological capital and promotive voice into the leadership–behavior relationship provides a more holistic theoretical perspective on employee agency. Ultimately, the proposed research advances theoretical discourse by offering a comprehensive account of how paradoxical leadership activates psychological and behavioral pathways that support adaptive, self-directed changes in employee roles.

### 6.2. Practical Implications

The findings derived from the proposed hypotheses offer valuable insights into managerial practice, particularly in shaping leadership strategies and fostering employee development. By demonstrating that paradoxical leadership enhances positive psychological capital and encourages job crafting through promotive voice, the findings suggest that organizations should consider adopting leadership approaches that reconcile competing demands. Leaders who exemplify such paradox-embracing behaviors may be better equipped to inspire psychological growth among employees, enabling them to develop greater confidence, optimism, and resilience in their roles. This study advances prior job crafting research by shifting the focus from individual differences and job characteristics to leadership as a critical antecedent. Specifically, it highlights paradoxical leadership as a contextual driver that cultivates psychological resources and promotive voice, thereby enabling employees to engage in job crafting.

Promoting paradoxical leadership can be instrumental in cultivating a safe work climate that motivates employees to initiate constructive changes and proactively reconfigure their tasks in alignment with organizational objectives (Madaan et al., 2025). In this context, promoting employee voice should not be viewed merely as a communication channel, but as a developmental pathway through which employees engage more deeply with their work and contribute meaningfully to organizational transformation (Ruck et al., 2017). By fostering promotive voice, organizations may unlock the potential of their workforce to not only adapt to change but also to drive it from within.

Furthermore, organizations should consider integrating leadership development initiatives that equip managers with the skills to navigate paradoxes while simultaneously supporting employees’ psychological resource building. These programs should emphasize the importance of nurturing positive psychological states and fostering an environment where employee input is genuinely valued and acted upon. Encouraging job crafting can also be institutionalized through flexible job designs and supportive performance management systems (Sun et al., 2025). Collectively, these practices can lead to a more engaged, proactive, and resilient workforce. Ultimately, the practical implications underscore the importance of leadership approaches that recognize and utilize individual psychological strengths, thereby promoting sustained organizational vitality and long-term success.

### 6.3. Limitations and Future Studies

Despite the theoretical and practical contributions of the research, several limitations should be acknowledged, which offer important directions for future research. First, the hypothesized relationships among paradoxical leadership, positive psychological capital, promotive voice, and job crafting are derived from theoretical logic and may not fully capture the dynamic and context-dependent nature of organizational behavior. The model reflects an idealized sequence of influences, yet real-world interactions among these constructs may be more reciprocal or cyclical. Future research would benefit from employing longitudinal or time-lagged designs to verify the temporal ordering of these relationships and to explore how they may unfold across different stages of employee development and organizational change.

Moreover, the mediating roles of positive psychological capital and promotive voice, while theoretically sound, may not fully account for the range of psychological and interpersonal processes through which paradoxical leadership impacts job crafting. Additional mediating constructs, such as psychological empowerment, perceived organizational support, or relational energy, could be explored in future studies to deepen our understanding of the indirect pathways involved. Furthermore, the current model does not account for potential moderating factors that may influence the strength or direction of these effects. For example, individual-level traits such as regulatory focus, self-efficacy, or resilience may shape how employees respond to paradoxical leadership, while organizational characteristics such as structure, culture, or team climate could enhance or constrain the emergence of promotive voice and job crafting behaviors.

Finally, the research assumes that the effects of paradoxical leadership are uniformly positive. However, employees may interpret paradoxical leadership behaviors differently depending on their personal values, past experiences, or expectations of leadership. What is perceived by one employee as a supportive and flexible approach may be viewed by another as ambiguous or inconsistent. Future research should investigate the boundary conditions under which paradoxical leadership fosters or inhibits psychological and behavioral outcomes. By addressing these limitations, future studies can refine the theoretical framework and contribute to a more nuanced and context-sensitive understanding of how paradoxical leadership influences employee thriving.

## Figures and Tables

**Table 1:** Confirmatory factor analysis.

Item	Estimate		S.E.	C.R.	AVE	CR
	β	B				
PL1	0.707	1			0.521	0.883
PL2	0.699	0.988	0.080	12.425		
PL3	0.727	1.032	0.080	12.920		
PL4	0.673	0.954	0.080	11.938		
PL5	0.749	1.060	0.080	13.252		
PL6	0.729	1.030	0.080	12.932		
PL7	0.685	0.969	0.080	12.150		
PL8	0.755	1.075	0.079	13.537		
PL9	0.668	0.946	0.080	11.896		
PL10	0.725	1.025	0.079	12.904		
PL11	0.720	1.021	0.080	12.807		
PL12	0.699	0.989	0.079	12.445		
PL13	0.790	1.120	0.080	14.004		
PL14	0.764	1.080	0.080	13.506		
PPC1	0.694	1			0.527	0.816
PPC2	0.684	0.977	0.085	11.545		
PPC3	0.788	1.135	0.085	13.314		
PPC4	0.695	0.999	0.085	11.755		
PPC5	0.723	1.043	0.085	12.216		
PPC6	0.718	1.037	0.085	12.196		
PPC7	0.749	1.088	0.085	12.812		
PPC8	0.748	1.078	0.085	12.694		
JC5	0.720	1			0.525	0.735
JC4	0.755	1.067	0.082	13.012		
JC3	0.728	1.011	0.081	12.444		
JC2	0.719	0.997	0.081	12.277		
JC1	0.701	0.978	0.081	12.072		
PV8	0.809	1			0.626	0.870
PV7	0.820	1.014	0.058	17.613		
PV6	0.788	0.975	0.059	16.482		
PV5	0.790	0.977	0.059	16.627		
PV4	0.775	0.958	0.059	16.187		
PV3	0.771	0.954	0.059	16.093		
PV2	0.779	0.965	0.059	16.329		
PV1	0.798	0.986	0.059	16.827		
Model fit	CMIN/DF = 1.131, *p* < 0.001, RMR = 0.066, GFI = 0.908, CFI = 0.990, TLI = 0.989, IFI = 0.990, RFI = 0.911, NFI = 0.918, RMSEA = 0.020.					

Notes: N = 340. PL = paradoxical leadership, PPC = positive psychological capital, JC = job crafting, PV = promotive voice.

**Table 2:** Comparison of measurement models.

Model	Factor Structure	χ^2^	df	χ^2^/df	NFI	IFI	TLI	RMSEA
4-factor	PL, PPC, JC, PV	301.126	264	1.131	0.918	0.99	0.989	0.02
3-factor	PL, PPC, (JC + PV)	968.451	365	1.354	0.901	0.972	0.97	0.032
2-factor	PL, (PPC + JC + PV)	1456.231	423	2.056	0.849	0.916	0.91	0.056
1-factor	(PL + PPC + JC + PV)	1969.688	560	3.517	0.741	0.8	0.786	0.086

Notes: PL = paradoxical leadership; PPC = positive psychological capital; JC = job crafting; PV = promotive voice. Parentheses indicate that constructs were combined into a single latent factor.

**Table 3:** Mean, standard deviations, and correlations.

Variables	Mean	S.D.	PL	PPC	JC	PV
PL	3.120	1.054	1			
PPC	3.215	1.083	0.586 **	1		
JC	3.830	1.115	0.655 **	0.653 **	1	
PV	3.636	1.162	0.646 **	0.665 **	0.687 **	1

Notes: N = 340, ** *p* < 0.01. PL = paradoxical leadership, PPC = positive psychological capital, JC = job crafting, PV = promotive voice.

**Table 4:** The results for hypotheses 1–4.

Hypothesized Path	Estimate	SE	CR	P
H1. PL → PPC	0.665	0.069	9.632	***
H2. PL → JC	0.266	0.062	4.329	***
H3. PPC → PV	0.824	0.079	10.469	***
H4. PV → JC	0.787	0.081	9.754	***
Model fit	CMIN/DF = 1.225, *p* < 0.001, CFI = 0.982, TLI = 0.981, IFI = 0.982, RFI = 0.904, NFI = 0.910, RMSEA = 0.026.			

Notes: N = 340, *** *p* < 0.001. PL = paradoxical leadership, PPC = positive psychological capital, JC = job crafting, PV = promotive voice.

**Table 5:** Mediating effects of PPC and PV.

Variables	H5		H6	
	Model 1	Model 2	Model 3	Model 4
	(PPC)	(PV)	(JC)	(JC)
Gender	0.299	0.251	0.044	0.131
Age	0.045	0.032	0.079	0.068
Position	0.214 *	0.191	0.046	0.131
PL	0.690 ***	0.801 ***	0.341 ***	0.780 ***
PPC			0.319 ***	
PV			0.530 ***	
R^2^	0.357	0.425	0.636	0.431
ΔR^2^	-	-	-	0.205
F value	46.557 ***	62.028 ***	96.970 ***	63.336 ***

Notes: N = 340, * *p* < 0.05, *** *p* < 0.001. PL = paradoxical leadership, PPC = positive psychological capital, JC = job crafting, PV = promotive voice.

**Table 6:** The test results of mediating effects.

Hypothesis	Effect	Boot SE	Boot LLCI	Boot ULCI
Total Effect	0.712	0.051	0.612	0.808
Total indirect effect	0.446	0.042	0.368	0.530
PL → PPC → JC	0.136	0.032	0.076	0.200
PL → PV → JC	0.189	0.032	0.132	0.258
PL → PPC → PV → JC	0.121	0.020	0.086	0.161

Notes: PL = paradoxical leadership, PPC = positive psychological capital, JC = job crafting, PV = promotive voice.

## Data Availability

The data supporting this study’s findings are available from the corresponding author upon reasonable request.
